# Newly-recorded species of the genus *Atkinsonia* Stainton, 1859 (Lepidoptera, Stathmopodidae) from Korea with checklist

**DOI:** 10.3897/BDJ.13.e173562

**Published:** 2025-11-18

**Authors:** In-Won Jeong, Sora Kim

**Affiliations:** 1 Jeonbuk National University, Jeonju, Republic of Korea Jeonbuk National University Jeonju Republic of Korea

**Keywords:** Stathmopodidae, *
Atkinsonia
*, checklist, new record

## Abstract

**Background:**

The genus *Atkinsonia* in the family Stathmopodidae comprises 13 species described worldwide and only one is recorded from the Korean Peninsula. Despite this low species richness, the genus shows broad ecological diversity at the larval stage, ranging from phytophagy to carnivory, which supports the need for a comprehensive taxonomic study.

**New information:**

We report *Atkinsonia beijingana* as a newly-recorded species from the Korean Peninsula. We describe the adult morphology and genitalia. A worldwide checklist of *Atkinsonia* species and their distributions is also provided to improve understanding of the genus.

## Introduction

The family Stathmopodidae, first described in 1913, is a family within the superfamily Gelechioidea, currently comprising approximately 400 species in 40 genera (van Nieukerken et al. 2011). While some species within this family are considered serious agricultural pests, the group exhibits ecological diversity with species feeding on moss, fern spores, aphids and other substrates depending on the species (Terada 2016). Despite this diversity, the small size and high interspecific similarity of these moths have made species identification challenging, limiting extensive research on the family (Kim et al. 2017). Research has been particularly scarce for genera other than *Stathmopoda*, which accounts for more than half of all described species in the family (Terada 2016).

Amongst these, *Atkinsonia*, described by Stainton (1859), shows diverse ecological characteristics despite its relatively low species diversity. This genus is mainly distributed in Asia and exhibits a wide range of ecological traits, from phytophagy on oak trees to carnivory on aphids (Morimoto and Shibao 1993, Shen et al. 2024). Carnivory is exceptionally rare within Lepidoptera, occurring in less than 0.13% of the approximately 200,000 species (Rubinoff et al. 2025). Only 13 species have been recorded within this genus, including two newly-described species in 2016 (Wang et al. 2016). On the Korean Peninsula, the carnivorous species *A. ignipicta* (Butler), which feeds on aphids during its larval stage, was first reported from South Korea in 1983 (Park et al. 1983); however, no additional species have been documented since then. In this study, we report the phytophagous species *A. beijingana* (Yang), which feeds on oak trees, from the Korean Peninsula for the first time, based on morphological characteristics and genital structure. We also review the distributional range of each species through a checklist of the genus.

## Materials and methods

**Collection**

Specimens were collected by sweeping during the day and by bucket traps (12 V/20 W) and light traps (220 V/200 W) at night. The collected materials were mounted on micropins with the thorax and wings spread and dried in an oven at 50°C for at least two weeks. The specimens were then double-mounted and transferred to storage boxes for preservation.

**Identification**

Specimens were identified in accordance with the dissection protocol of Kim et al. (2017). All dissections were conducted using a Leica EZ4 stereomicroscope (Leica, Germany). Adult and genitalia were imaged on a Leica S8 APO stereomicroscope (Leica, Germany), fitted with a Tucsen Dhyana 400 DC digital camera (Tucsen, China) and illuminated by a Leica LED 5000 HDI dome light (Leica, Germany). Focus-stacked composites were produced with Mosaic (Tucsen, China) and Helicon Focus (Helicon Soft, Ukraine) and images received minor adjustments in Adobe Photoshop 2024 (Adobe, USA).

**FE-SEM (Field Emission-Scanning Electron Microscope) imaging**

To elucidate fine structures beyond naked-eye resolution, we imaged the frenulum with FE-SEM. Specimens were mounted on SEM stubs using 5 mm conductive carbon double-sided tape (Nissin EM Co., Japan). After evacuation with an Adixen 2005 SD vacuum pump (PFEIFFER VACUUM, Germany), a platinum layer was deposited for 90 s with a Leica EM ACE200 (Leica, Germany). Samples were analyed by Gemini 500 FE-SEM (Carl Zeiss, Germany) installed in the Center for University-wide Research Facilities (CURE), at Jeonbuk National University, with stage positioning and orientation managed in ZEN core Documentation (Carl Zeiss, Germany).

## Taxon treatments

### Atkinsonia beijingana

(Yang, 1977)

24C6E3B6-7963-590C-A73F-5707A3FE97D7

Oedematopoda beijingana Yang 1977: 148. Type locality: China (Beijing).
Atkinsonia beijingana (Yang): Sinev (2015): 22.

#### Materials

- **Type status:** Other material. **Occurrence:** catalogNumber: IPE JBNU-13094; recordedBy: Bae et al.; individualCount: 1; sex: male; lifeStage: adult; occurrenceID: 010B4C70-6B5D-5D98-A5DB-4ED346C3AEE1; **Taxon:** scientificName: Atkinsonia beijingana; kingdom: Animalia; phylum: Invertebrate; class: Insecta; order: Lepidoptera; family: Stathmopodidae; genus: Atkinsonia; **Location:** country: Korea; stateProvince: Gyeonggi; locality: Mt. Soyo, Dongducheon-si; **Identification:** identifiedBy: In-Won Jeong; **Event:** eventDate: 17/05/1997; **Record Level:** language: en
- **Type status:** Other material. **Occurrence:** catalogNumber: IPE JBNU-13095; recordedBy: Bae et al.; individualCount: 1; sex: female; lifeStage: adult; occurrenceID: 9C8CACC2-996C-554B-9251-09D464914378; **Taxon:** scientificName: Atkinsonia beijingana; kingdom: Animalia; phylum: Invertebrate; class: Insecta; order: Lepidoptera; family: Stathmopodidae; genus: Atkinsonia; **Location:** country: Korea; stateProvince: Gyeonggi; locality: Mt. Soyo, Dongducheon-si; **Identification:** identifiedBy: In-Won Jeong; **Event:** eventDate: 17/05/1997; **Record Level:** language: en
- **Type status:** Other material. **Occurrence:** catalogNumber: IPE JBNU-13114; recordedBy: Chae et al.; individualCount: 1; sex: female; lifeStage: adult; occurrenceID: 4F3403F5-AC5D-5D2A-846F-AAFAED2690AE; **Taxon:** scientificName: Atkinsonia beijingana; kingdom: Animalia; phylum: Invertebrate; class: Insecta; order: Lepidoptera; family: Stathmopodidae; genus: Atkinsonia; **Location:** country: Korea; stateProvince: Jeollanam; locality: Gwangam-ri, Haebo-myeon, Hampyeong-gun; verbatimCoordinates: 35°11'N 126°31'E; **Identification:** identifiedBy: In-Won Jeong; **Event:** eventDate: 11/06/2010; **Record Level:** language: en
- **Type status:** Other material. **Occurrence:** catalogNumber: IPE JBNU-13085; recordedBy: Jiseung Kim; individualCount: 1; sex: male; lifeStage: adult; occurrenceID: 9E8B9923-60C9-54B2-8FB1-D744429F61ED; **Taxon:** scientificName: Atkinsonia beijingana; kingdom: Animalia; phylum: Invertebrate; class: Insecta; order: Lepidoptera; family: Stathmopodidae; genus: Atkinsonia; **Location:** country: Korea; stateProvince: Jeollanam; locality: Wonbongbanwol-gil 297, Gwangju-si; verbatimCoordinates: 35°12'N 126°50'E; **Identification:** identifiedBy: In-Won Jeong; **Event:** eventDate: 24/04/2023; **Record Level:** language: en
- **Type status:** Other material. **Occurrence:** catalogNumber: IPE JBNU-13223; recordedBy: Wonbin Lim; individualCount: 1; sex: male; lifeStage: adult; occurrenceID: F269B7E9-3203-5FD4-83B8-490E4E9AB3D2; **Taxon:** scientificName: Atkinsonia beijingana; kingdom: Animalia; phylum: Invertebrate; class: Insecta; order: Lepidoptera; family: Stathmopodidae; genus: Atkinsonia; **Location:** country: Korea; stateProvince: Jeollanam; locality: Mongtan myeon, Muan-gun; **Identification:** identifiedBy: In-Won Jeong; **Event:** eventDate: 17/05/2023; **Record Level:** language: en

#### Description

**Adult** (Fig. 1a, b, c). Wing expanse 11.0-12.0 mm. Head. Frons dark grey; vertex deep purple; occiput dark grey. Antenna deep purple with ocherous cilia. Labial palpus slightly upcurved; first segment ochre; second segment pale ochrous grey at the junction with first segment, overall greyish-purple; third segment dark greyish-purple with ochrous purple at the apex. Tongue ventrally greyish-ochre, dorsally pale grey. Thorax. Tegula orange. Thorax orange; prothorax dark grey; mesothorax with pale greyish-silver at caudal margin; metathorax dark grey, pale ochre at the caudal margin. Abdomen dark purple; second segment with ochrous white scales caudally; fifth segment dorsally white. Wing. Fore-wing entirely vermilion with grey base, bright vermilion at the edge of the apex; fringes fuscous, few bright vermilion at the apex. Hind-wing fuscous with dark ochrous base; in male, one frenulum originating from four pairs of strands; in female, three frenula originating from each strand; fringes fuscous. **Male genitalia** (Fig. 1d, e). Uncus blunt with numerous setae on the lateral margin; apically bifurcate and sharp. Gnathos absent. Valva broad, gently bent twice with round apex, slightly wider from the first bent part to the second bent part, narrower from the second bent part to the apex; costa round dorsally; cucullus bean-shaped with numerous setae, slightly shorter than twice the length of uncus; sacculus sclerotised, with ventrally long setae. Vinculum short and thick with blunt apex. Juxta round dorsally with very gently vertices. Anellar lobes developed, club-shaped. Aedeagus approximately twice longer than uncus; cornutus absent; from mid-point to apex narrower and sclerotised, other weakly sclerotised; weakly sclerotised rectangular plate near base; apical patch of stimuli sclerotised, with acute apex. **Female genitalia** (Fig. 1f). Papillae anales length ca. 1.3 times longer than wide, weakly sclerotised, with numerous short setae. Eighth abdominal segment weakly sclerotised, cephalodorsally round, with long setae irregularly arranged along the caudal margin. Apophyses posteriores slightly 1.5 times longer than apophyses anteriores. Ostium bursae cup-shaped, crescent-shaped sclerotised structure inner. Ductus bursae narrow, slightly longer than half of the corpus bursae. Corpus bursae with two crescent-shaped signa near the caudal margin. Ductus seminalis very narrow and long, originating at about half of ductus bursae with microspines overall; bulla small, at about 2/5 of ductus seminalis.

#### Diagnosis

This species is similar to *A. ignipicta*, but has differences in the marking of the fore-wing and structures of the genitalia. In *A. ignipicta*, the fore-wing is dark red with a black posterior margin and in the male genitalia, the costa is angled dorsally and the anellar lobes are oval-shaped. However, in this species, the fore-wing is entirely dark red and, in the male genitalia, the dorsal margin of the costa is rounded and the anellar lobes are club-shaped.

## Checklists

### Checklist of the genus *Atkinsonia* Stainton, 1859

#### Atkinsonia beijingana

(Yang, 1977)

02F8368D-2CAD-5D8D-99BE-82D6E2A8C19D

Oedematopoda beijingana Yang 1977: 148. Type locality: China (Beijing).
Atkinsonia beijingana (Yang): Sinev (2015): 22.

##### Feeds on

*Castanea crenata* Siebold and Zucc., *Castanopsis kawakamii* Hayata, *Quercus acutissima* Carruth. and *Q. serrata* Murray (Fagaceae) (Terada 2016; Shen et al. 2024).

##### Distribution

Korea (this study; Gyeonggi, Jeollanam), Japan (Aichi, Fukuoka, Hyogo, Kagoshima, Kyoto, Nara, Osaka, Wakayama, Yamaguchi), China (Beijing, Guangxi, Guizhou, Hainan, Hebei, Henan, Shaanxi, Tianjin, Zhejiang) (Terada 2016; Wang et al. 2016; Yagi et al. 2019).

#### Atkinsonia brevisaccula

Wang, Guang and Sinev, 2016

1EE2EF0B-D994-5E42-832D-5D9239F41B12

Atkinsonia brevisaccula Wang et al. 2016: 4208. Type locality: China (Hainan).

##### Feeds on

Unknown.

##### Distribution

China (Hainan) (Wang et al. 2016).

#### Atkinsonia butalistis

(Strand, 1917)

ED355722-ECF1-5BB5-A524-95B5ADEB76FC

Oedematopoda butalistis Strand 1917: 152. Type locality: China (Taiwan).
Atkinsonia butalistis (Strand): Wang et al. (2016): 4208.

##### Feeds on

Unknown.

##### Distribution

Chian (Taiwan) (Wang et al. 2016).

#### Atkinsonia clerodendronella

Stainton, 1859

85E0F25B-7D72-5262-9761-789D0797FC67

Atkinsonia clerodendronella Stainton 1859: 125. Type locality: India (West Bengal).
Oedematopoda clerodendronella (Stainton): Walsingham (1889): 20.

##### Feeds on

*Clerodendrum infortunatum* L. and *Anisomeles indica* (L.) Kuntze (Lamiaceae) (Maxwell-Lefroy and Howlett 1909; Fletcher 1920).

##### Distribution

China (Guizhou), India (Bihar, West Bengal, Kerala) (Fletcher 1920; Wang et al. 2016; Shamsudeen and Pathania 2022; Sahoo 2025).

#### Atkinsonia cypris

(Meyrick, 1905)

30297712-5707-5AB2-84F2-B8302DBAD3D1

Oedematopoda cypris Meyrick 1905: 608. Type locality: Sri Lanka (Kandy).
Atkinsonia cypris (Meyrick): Sinev (2015): 22.

##### Feeds on

*Kerria albizziae* (Green) (Hemiptera, Kerriidae) (Meyrick 1905).

##### Distribution

Sri Lanka (Kandy) (Meyrick 1905)

#### Atkinsonia flammifera

(Meyrick, 1915)

68EEDD69-CAD8-52A7-B3B0-9AA68DCF21C7

Oedematopoda flammifera Meyrick 1915: 338. Type locality: India (Bihar).
Atkinsonia flammifera (Meyrick): Sinev (2015): 22.

##### Feeds on

*Mangifera indica* L. (Anacardiaceae) (Fletcher 1920).

##### Distribution

India (Bihar) (Meyrick 1915).

#### Atkinsonia furcata

(Wang, 2008)

34E77FF9-41A8-524A-A244-14FE26A85E3A

Oedematopoda furcata Wang and Sun 2008: 37. Type locality: China (Fujian).
Atkinsonia furcata (Wang): Wang et al. (2016): 4208.

##### Feeds on

Unknown.

##### Distribution

China (Fujian) (Wang et al. 2016).

#### Atkinsonia ignipicta

(Butler, 1881)

B922C8AB-AB0F-59C8-B81A-8FFE93BF70AF

Eretmocera ignipicta Butler 1881: 593. Type locality: Japan (Honshu).
Oedematopoda ignipicta (Butler): Walsingham (1889): 22-23.
Oedematopoda nohirai Matsumura 1931: 1011.
Oedematopoda semirubra Meyrick 1936: 619.
Atkinsonia ignipicta (Butler): Kasy (1976): 429.

##### Feeds on

*Ceratovacuna japonica* (Takahashi) (Hemiptera, Aphididae) and *Pseudoregma bambucicola* (Takahashi) (Hemiptera, Pemphigidae) (Moriuti 1982; Morimoto and Shibao 1993).

##### Distribution

Korea (Gyeonggi), Japan (Hokkaido, Nagano, Gifu, Wakayama, Ehime), China (Guangxi, Guizhou, Hainan) (Park et al. 1983; Terada 2016; Wang et al. 2016).

#### Atkinsonia ignispergens

(Diakonoff, 1948)

81B48148-3F5C-5A72-891E-F9B0EBF5E7BB

Snellenia ignispergens Diakonoff 1948: 271. Type locality: Japan.
Atkinsonia ignispergens (Diakonoff): Sinev (2015): 22.

##### Feeds on

Unknown.

##### Distribution

Japan (Diakonoff 1948).

#### Atkinsonia parignipicta

Wang, Guang and Sinev, 2016

CDEB2E90-4B0D-59B8-A343-DADE1A506186

Atkinsonia parignipicta Wang et al. 2016: 4208. Type locality: China (Hainan).

##### Feeds on

Unknown.

##### Distribution

China (Henan) (Wang et al. 2016).

#### Atkinsonia pyromyia

(Meyrick, 1929)

E668D374-0825-5D01-B2E4-1533D0457604

Oedematopoda pyromyia Meyrick 1929: 542. Type locality: India (Meghalaya).
Atkinsonia pyromyia (Meyrick): Sinev (2015): 22.

##### Feeds on

*Oregma* spp. (Hemiptera, Aphididae) (Fletcher 1933).

##### Distribution

India (Meghalaya) (Meyrick 1929).

#### Atkinsonia swetlanae

Sinev, 1988

F8CA997C-BF57-5D03-83AA-B90EB6EC77EE

Atkinsonia swetlanae Sinev 1988: 120. Type locality: Russia (Primorsky Territory).
Oedematopoda jiyuanica Wang and Sun 2008: 36.

##### Feeds on

Unknown.

##### Distribution

China (Henan, Hubei, Shanxi, Sichuan, Zhejiang), Russia (Far East) (Wang et al. 2016).

#### Atkinsonia venusta

(Meyrick, 1913)

4069B79A-6DDE-5FF9-B071-1F6089F8B3FF

Oedematopoda venusta Meyrick 1913: 97. Type locality: India (Madhya Pradesh).
Atkinsonia venusta (Meyrick): Sinev (2015): 23.

##### Feeds on

*Kerria lacca* (Kerr) (Hemiptera, Kerriidae) (Meyrick 1913).

##### Distribution

India (Madhya Pradesh) (Meyrick 1913).

## Supplementary Material

XML Treatment for Atkinsonia beijingana

XML Treatment for Atkinsonia beijingana

XML Treatment for Atkinsonia brevisaccula

XML Treatment for Atkinsonia butalistis

XML Treatment for Atkinsonia clerodendronella

XML Treatment for Atkinsonia cypris

XML Treatment for Atkinsonia flammifera

XML Treatment for Atkinsonia furcata

XML Treatment for Atkinsonia ignipicta

XML Treatment for Atkinsonia ignispergens

XML Treatment for Atkinsonia parignipicta

XML Treatment for Atkinsonia pyromyia

XML Treatment for Atkinsonia swetlanae

XML Treatment for Atkinsonia venusta

## Figures and Tables

**Figure 1a.**
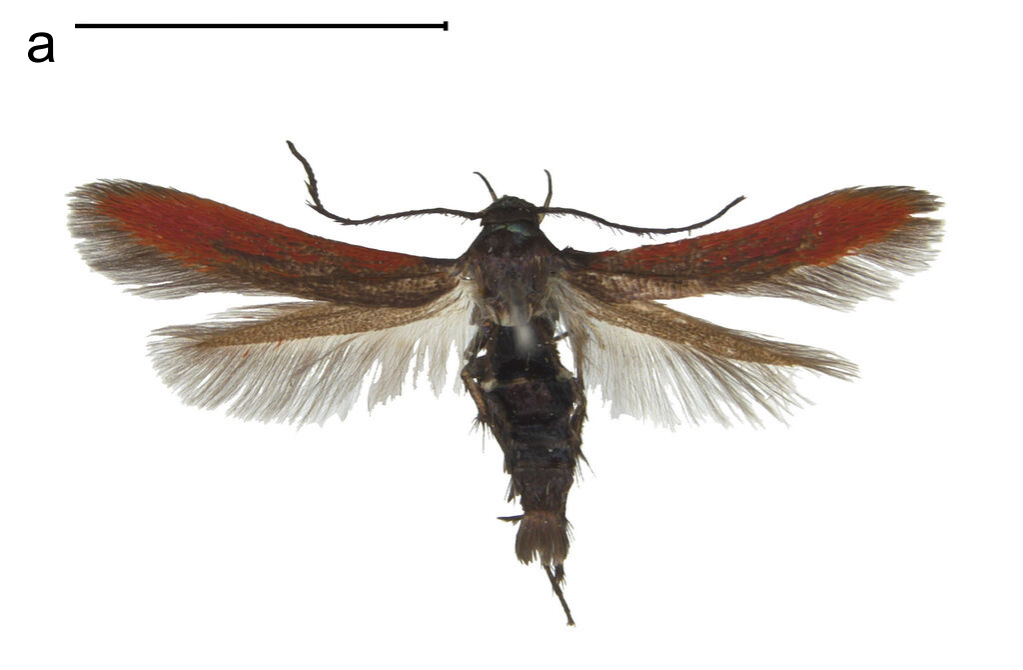
Adult. Scale bar: 5.0 mm;

**Figure 1b.**
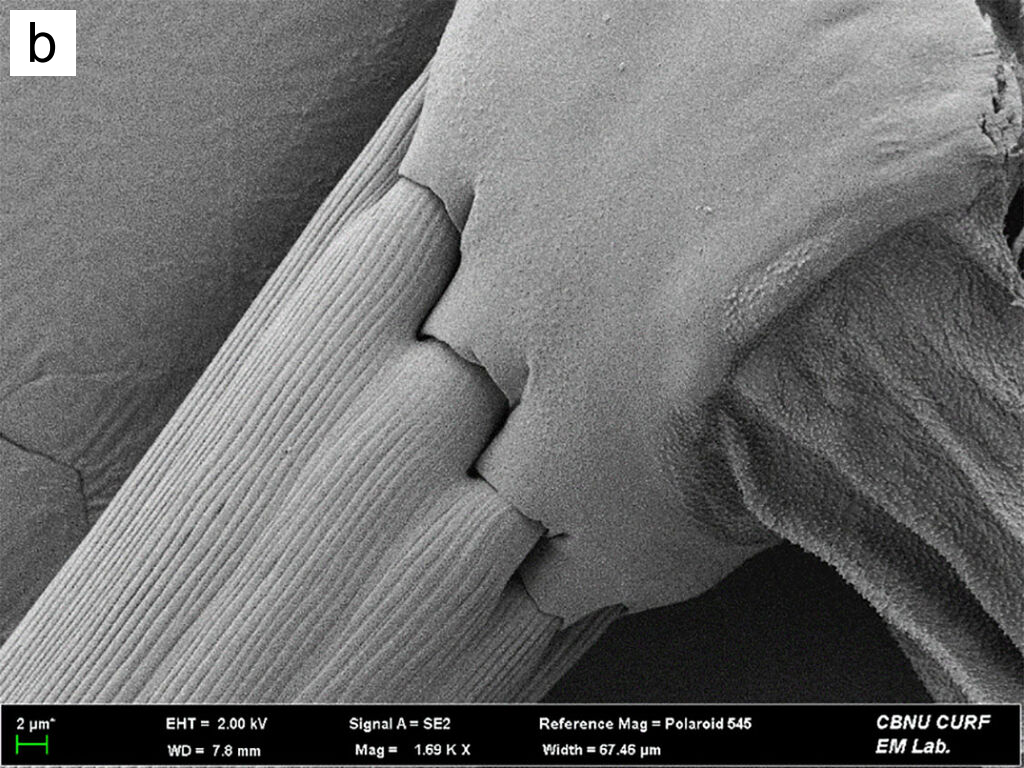
Frenulum of the male;

**Figure 1c.**
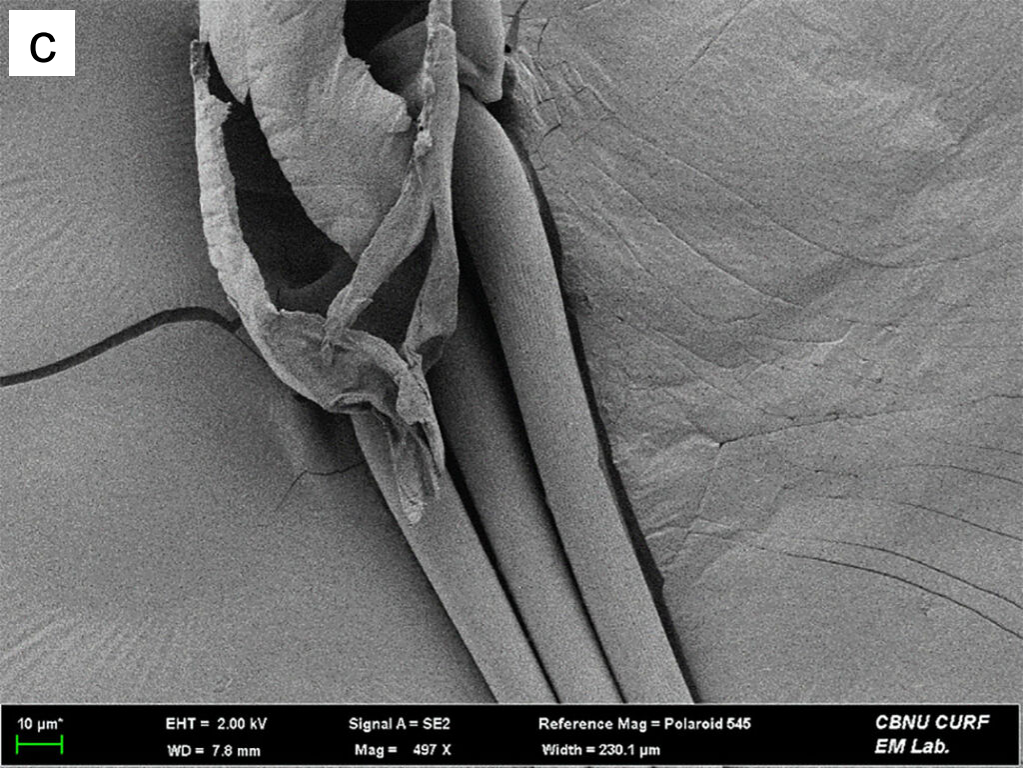
Frenula of the female;

**Figure 1d.**
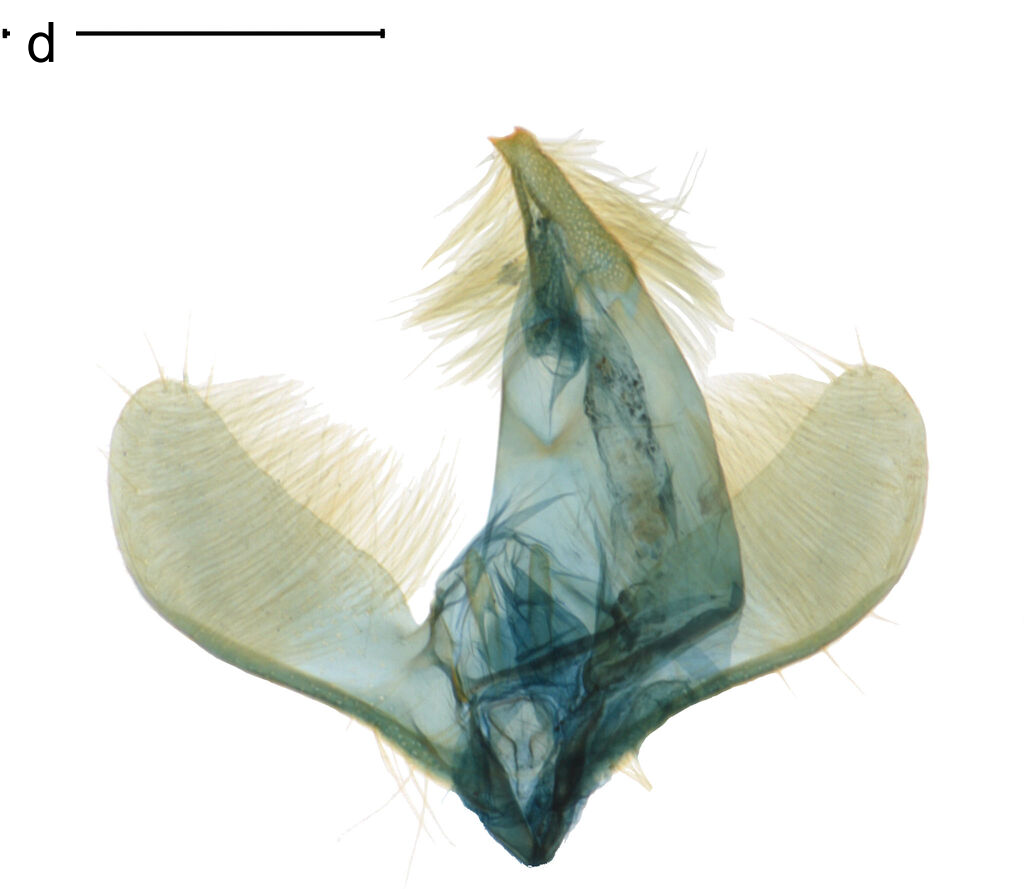
Male genitalia. Scale bar: 0.5 mm;

**Figure 1e.**
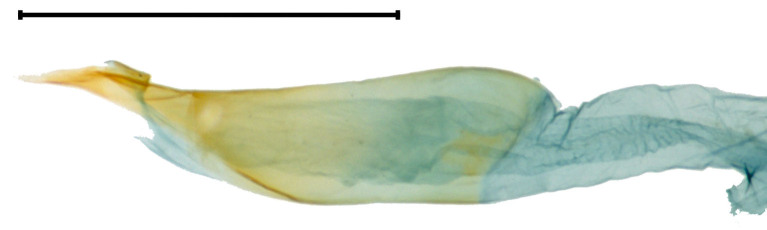
Aedeagus. Scale bar: 0.5 mm;

**Figure 1f.**
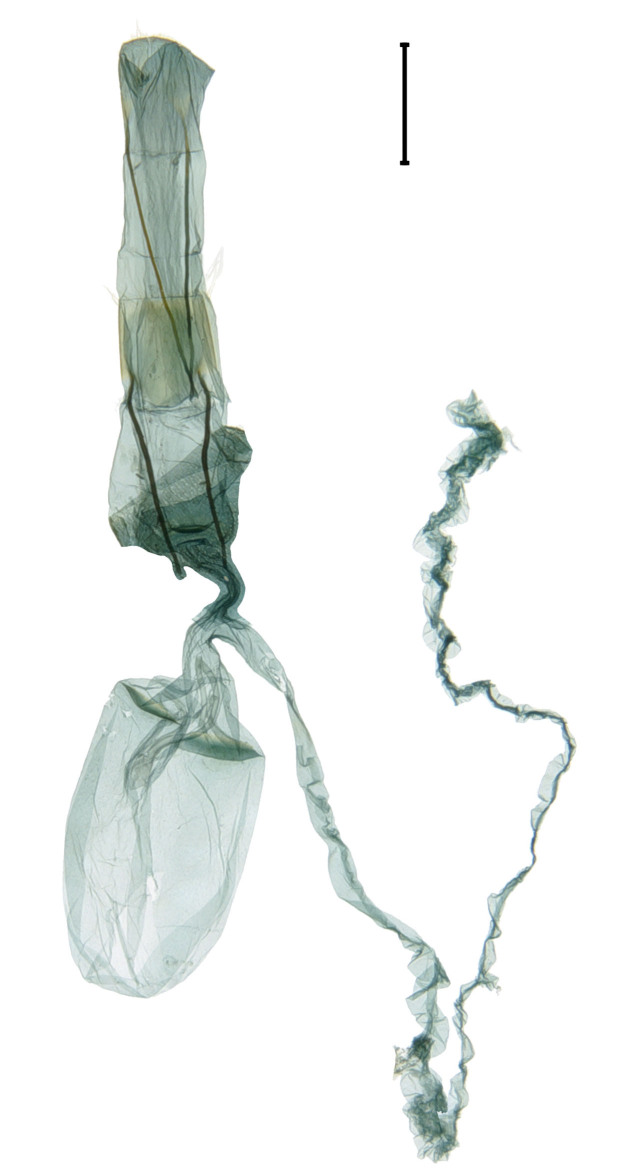
Female genitalia. Scale bar: 0.5 mm.
